# Sources of Added Sugars Intake Among the U.S. Population: Analysis by Selected Sociodemographic Factors Using the National Health and Nutrition Examination Survey 2011–18

**DOI:** 10.3389/fnut.2021.687643

**Published:** 2021-06-17

**Authors:** Laurie Ricciuto, Victor L. Fulgoni, P. Courtney Gaine, Maria O. Scott, Loretta DiFrancesco

**Affiliations:** ^1^Department of Nutritional Sciences, University of Toronto, Toronto, ON, Canada; ^2^Nutrition Impact, LLC, Battle Creek, MI, United States; ^3^The Sugar Association, Inc., Washington, DC, United States; ^4^Source! Nutrition, Toronto, ON, Canada

**Keywords:** added sugars, food sources, sociodemographics, NHANES, US

## Abstract

Recent estimates of added sugars intake among the U.S. population show intakes are above recommended levels. Knowledge about the sources of added sugars contributing to intakes is required to inform dietary guidance, and understanding how those sources vary across sociodemographic subgroups could also help to target guidance. The purpose of this study was to provide a comprehensive update on sources of added sugars among the U.S. population, and to examine variations in sources according to sociodemographic factors. Regression analyses on intake data from NHANES 2011–18 were used to examine sources of added sugars intake among the full sample (*N* = 30,678) and among subsamples stratified by age, gender, ethnicity, and income. Results showed the majority of added sugars in the diet (61–66%) came from a few sources, and the top two sources were sweetened beverages and sweet bakery products, regardless of age, ethnicity, or income. Sweetened beverages, including soft drinks and fruit drinks, as well as tea, were the largest contributors to added sugars intake. There were some age-, ethnic-, and income-related differences in the relative contributions of added sugars sources, highlighting the need to consider sociodemographic contexts when developing dietary guidance or other supports for healthy eating.

## Introduction

Recommendations on the intake of added sugars are generally used to inform population-wide dietary guidance. The Institute of Medicine has suggested a maximum daily limit of 25% of calories from added sugars (1). More recent recommendations are lower, with the World Health Organization issuing a guideline of <10% of calories per day from “free sugars” (inclusive of both added sugars and sugars naturally present in 100% fruit juice) and a conditional recommendation for a further reduction to <5% (2). The 2020–2025 Dietary Guidelines for Americans recommend limiting foods and beverages higher in added sugars, and that a healthy dietary pattern limits added sugars to <10% of calories (3).

Estimates of added sugars intake for the U.S. population show that recent intake levels are above current recommendations; however, they are lower than previous intakes: population-wide (2+ years) daily average added sugars intake was approximately 18% of calories in 1999 to 2000 (4) compared to 13% among individuals 1+ years in 2015 to 2016 (5). However, examining added sugars intake levels without considering the sources of added sugars is not sufficient to inform dietary guidance. Furthermore, understanding how added sugars in the diet may vary across sociodemographic subgroups could also help to develop more targeted dietary guidance.

Sources of added sugars and sociodemographic factors have been examined in conjunction with added sugars intake. Such investigations have been carried out for all age groups across the U.S. population using NHANES 2009–12 (6) and for younger age groups using NHANES 2011–14 (7, 8); and results from these studies have shown that sweetened beverages and sweet bakery products are the top two dietary sources of added sugars. Based on later NHANES 2013–16 data, sweetened beverages remain the top source for most age groups (9). However, recent studies of added sugars sources and sociodemographic factors other than age and gender are limited. Factors such as ethnicity and income have been examined previously (data from 1994 to 2010) but mainly in children (10–12) or other specific cohorts (13, 14).

The purpose of the present study was to provide a comprehensive update on sources of added sugars intake among the U.S. population using NHANES 2011–18 and to examine variations in added sugars sources according to the selected sociodemographic factors: age; gender; ethnicity; and, income.

## Materials and Methods

### Data Source and Participants

The health and nutritional status of the U.S. population is monitored regularly through the NHANES, a cross-sectional survey conducted by the Centers for Disease Control and Prevention National Center for Health Statistics (15). The survey sample is selected through a clustered stratified multistage sampling design, with periodic oversampling of select population groups, and is representative of the non-institutionalized civilian resident population. Data collection for the dietary interview component of NHANES, called What We Eat in America (WWEIA), is conducted by the U.S. Department of Agriculture (USDA) Food Surveys Research Group (FSRG) (16). Dietary interviews are conducted by trained interviewers using the Automated Multiple Pass Method: for children 2–5 years, diet interviews are conducted with a proxy; those 6–11 years receive help from a proxy; and, individuals 12+ years complete the interviews themselves. The first 24-h dietary recall interview is conducted in person and a second 24-h recall is conducted by telephone 3 to 10 days later on a subsample of participants. Details of the NHANES survey design and dietary data collection procedures are reported elsewhere (15, 16).

We used data from four cycles of NHANES (2011–12, 2013–14, 2015–16, and 2017–18) to provide large enough sample sizes for specific groups. Analyses were conducted of added sugars sources among all age groups (2–8, 9–18, 19–50, 51–70, and 71+ years) and by gender, ethnicity, and income for those 2–18 and 19+ years.

NHANES 2011–18 procedure and protocols (#2011–17, #2018–01) were reviewed and approved by the NCHS Research Ethics Review Board. Ethical review and approval were waived for this study, due to the use of secondary data. NHANES obtained written, informed consent for all adult participants.

### Added Sugars Intake and Sources

The Food Patterns and Equivalents Database (FPED), developed by the USDA FSRG, converts foods and beverages reported in the 24-h recalls into food pattern equivalents corresponding to those in the Dietary Guidelines for Americans (17). The “added sugars” food pattern component is comprised of caloric sweeteners (syrups, sugars, and others) using the definition of added sugars as “sugars that are added to foods as an ingredient during preparation, processing, or at the table; added sugars do not include naturally occurring sugars such as lactose present in milk and fructose present in whole or cut fruit and 100% fruit juice” (17), which are similarly defined for the nutrition labeling of food (18). While this definition of added sugars has been stable over time, fruit juice concentrates not diluted to single strength juices have been designated as added sugars since 2011–12, which may affect added sugars values for foods such as snack bars, ready-to-eat (RTE) cereals, baby foods, and fruit spreads. More detail on determining added sugars content of foods is provided in FPED documentation (17).

We determined added sugars intake using the FPED for each NHANES cycle. Day 1 intake data were used as these data were collected in person; whereas day 2 intakes were collected by telephone, and a validation of this method, to our knowledge, has never been reported. Furthermore, a single day of intake is sufficient for providing an accurate estimate of population mean intake (19), which was required for our analyses. Mean added sugars intake as a percentage of total calories was calculated for each age group (2–8, 9–18, 19–50, 51–70, and 71+ years) and for two overall age groups (2–18 and 19+ years) using the population ratio method, which required summing the daily added sugars intake for all individuals in a particular age group, and then dividing by the sum of daily calorie intake for the same individuals. The population ratio method was used because it provides an unbiased estimate of population intakes when using 1 day of intake data (20).

In order to facilitate analyses of population food intakes, USDA uses a food grouping scheme for WWEIA, in which foods and beverages are grouped according to their similar nutrient content and common use in the diet; and individual food categories can be combined into larger food groups if required for analytical purposes (21). We analyzed sources of added sugars based on the USDA/WWEIA food categories (Table 1). Within each of the food categories and larger food groups, mean added sugars contribution expressed as grams was determined, and then percentage of total daily added sugars intake was calculated using the population ratio method; food sources were then ranked from highest to lowest.

**Table 1:** Breakdown of food groups into types of foods (categories^a^) that provide added sugars.

**Food group**	**Food category**
Breads, Rolls, Tortillas	Yeast breads; Rolls and buns; Bagels and English muffins; Tortillas
Candy	Candy: containing chocolate; not containing chocolate
Coffee and Tea	Coffee; Tea
Fats and Oils	Butter and animal fats; Margarine; Cream cheese, sour cream, whipped cream; cream and cream substitutes; Mayonnaise; Salad dressings and vegetable oils
Flavored Milk	Flavored milk: whole; reduced fat; lowfat; non-fat
Other Desserts	Ice cream and frozen dairy desserts; Pudding; Gelatins, ices, sorbets
Quick Breads and Bread Products	Biscuits, muffins, quick breads; Pancakes, waffles, French toast
Ready-to-Eat Cereals	RTE^b^ cereal: higher sugar (>21.2 g/100 g); lower sugar (<21.2 g/100 g)
Sugars	Sugars and honey; Sugar substitutes; Jams, syrups, toppings
Sweet Bakery Products	Cakes and pies; Cookies and brownies; Doughnuts, sweet rolls, pastries
Sweetened Beverages	Soft drinks; Fruit drinks; Sport and energy drinks; Nutritional beverages; Smoothies and grain drinks
Yogurt	Yogurt: regular; Greek

a *What we eat in America food categories (21)*.

b *RTE, ready-to-eat*.

### Statistical Analyses

Data were analyzed using SAS 9.4 (SAS Institute, Cary, NC, USA), and weighting factors provided by NHANES were applied to adjust for the complex survey sampling design (primary sampling units and strata), non-response rates, and oversampling of certain subgroups (day 1 sample weights). Food sources contributing at least 2% to total daily added sugars intake based on NHANES 2011–12, the reference year, were considered for analysis. Linear regression analyses were used to compare mean added sugars intake from food sources in 2017–18 vs. 2011–12 for each age group, examining both the WWEIA food categories and the larger food groups. Means ± standard errors (SE) were used to present results, which is how NHANES data are typically presented. Given the large sample size used for these analyses and to help ensure extremely small differences were not deemed significant, a more conservative *p*-value of *p* < 0.01 was selected.

Additionally, the combined sample (2011–18) was stratified by gender, ethnicity (using the groups as self-defined in NHANES as Asian, Black, Hispanic, and White), and income [household poverty income ratio (PIR) of low, medium, and high (PIR<1.35, 1.35<PIR<1.85, and PIR>1.85, respectively)]. These PIR categories were chosen because similar percentages are used by the U.S. government to determine eligibility for federal programs, such as nutrition assistance and school lunch programs, with the higher values having higher socioeconomic status. Added sugars sources were then examined by gender for each age group, and by ethnicity and PIR for the two overall age groups (2–18 and 19+ years). Again, food sources contributing at least 2% to total daily added sugars intake based on NHANES 2011–12 (the reference year) were considered for analysis and regression analyses were used to compare mean added sugars intake from the WWEIA food categories sources in 2017–18 vs. 2011–12 for each age group using *p* < 0.01.

## Results

Using data from all four cycles of NHANES (2011–18), after exclusions for unreliable dietary data (*n* = 5,548) as determined by the USDA FSRG, pregnant or lactating females (*n* = 359), and kilocalories = 0 (*n* = 3), the final sample size was 30,678. Distributions of sociodemographic characteristics among the final sample are shown in Table 2. The final sample size using data from NHANES 2011–12 was 7,862, and from NHANES 2017–18 was 7,035.

**Table 2:** Distribution of sociodemographic characteristics for the combined NHANES 2011–18 analytical sample.

**Age and gender^a^ (total sample** ***N*** **=** **30,678)**					
**Age**	***n***	**% (SE)^b^**	**Gender**	***n***	**% (SE)**
2–8 years	4,759	9.40 (0.28)	Male	2,408	51.86 (1.01)
			Female	2,351	48.14 (1.01)
9–18 years	6,154	13.77 (0.31)	Male	3,075	50.14 (1.05)
			Female	3,079	49.86 (1.05)
19–50 years	10,286	42.34 (0.71)	Male	5,122	51.61 (0.65)
			Female	5,164	48.39 (0.65)
51–70 years	6,649	25.57 (0.58)	Male	3,251	47.51 (0.77)
			Female	3,398	52.49 (0.77)
71+ years	2,830	8.92 (0.32)	Male	1,415	43.95 (1.07)
			Female	1,415	56.05 (1.07)
**Ethnicity (total sample** ***N*** **=** **29,177^c^)**					
**Age**		**Ethnicity**		***n***	**% (SE)**
2–18 years (*n* = 10,143)		Asian		1,053	4.67 (0.46)
		Black		2,736	13.79 (1.35)
		Hispanic		3,356	23.91 (1.92)
		White		2,998	51.90 (2.49)
19+ years (*n* = 19,034)		Asian		2,282	5.53 (0.50)
		Black		4,528	11.41 (0.98)
		Hispanic		4,734	15.06 (1.18)
		White		7,490	64.53 (1.71)
**Income (total sample** ***N*** **=** **28,055^c^)**					
**Age**		**PIR^d^**		***n***	**% (SE)**
2–18 years (*n* = 10,082)		Low		4,588	35.29 (1.70)
		Medium		1,278	11.54 (0.69)
		High		4,216	53.17 (1.86)
19+ years (*n* = 17,973)		Low		6,123	24.26 (0.97)
		Medium		2,226	9.94 (0.45)
		High		9,624	65.80 (1.21)

a *Values for gender are within each age group*.

b *SE, standard error*.

c *Total sample sizes for ethnicity and income are different and lower than the total sample size for age and gender due to missing information for some individuals (ethnicity and/or income not reported)*.

d *PIR, poverty income ratio; low (PIR < 1.35), medium (1.35 < PIR < 1.85), high (PIR > 1.85)*.

In 2017–18, the population mean intake (SE) of added sugars as a percentage of calories was 12.7% (0.3), corresponding to a mean of 67.8 g/day (1.6). Intakes were highest among adolescents and teens 9–18 years at 14.3% (0.3), and lowest among older adults 71+ years at 11.3% (0.4), corresponding to a mean of 73.1 g/day (1.8) and 53.8 g/day (1.5) of added sugars, respectively.

### Children 2–8 Years

The top two sources of added sugars among children in 2017–18 were sweetened beverages and sweet bakery products, contributing 22.8 and 19.2%, respectively, to total daily added sugars intake (Table 3). Candy, RTE cereals, and other desserts ranked number three to five, and all together two-thirds (66%) of daily added sugars intake came from the top five food groups. The contribution from sweetened beverages was significantly lower in 2017–18 compared to 2011–12, while the contribution from sweet bakery products was significantly higher; however, they remained the top two sources of added sugars. The contribution from sugars was significantly lower in 2017–18 compared to 2011–12, and it fell in rank from number six to number eight.

**Table 3:** Food group sources^a^ and ranking of added sugars as a percentage of total daily added sugars intake among children 2–8 years, NHANES 2017–18 (*n* = 914) compared to NHANES 2011–12 (*n* = 1,436); values are mean (standard error) based on first day dietary recall.

**Food group**	**2017–18**		**2011–12**		***P*-value^b^**
	**% Added sugars from food group**	**Rank**	**% Added sugars from food group**	**Rank**	
Sweetened beverages	22.81 (1.63)	1	29.31 (1.48)	1	0.0032
Sweet bakery products	19.18 (1.03)	2	15.41 (0.81)	2	0.0040
Candy	9.52 (1.03)	3	7.04 (0.92)	4	0.0739
Ready-to-eat cereals	7.23 (0.65)	4	6.62 (0.40)	5	0.4239
Other desserts	7.08 (0.76)	5	7.73 (0.95)	3	0.5897
Flavored milk	6.14 (0.72)	6	6.25 (0.76)	7	0.9140
Coffee and tea	3.46 (1.08)	7	2.89 (1.03)	9	0.6979
Sugars	3.34 (0.38)	8	6.29 (0.93)	6	0.0031
Quick breads and bread products	2.96 (0.57)	9	2.29 (0.55)	10	0.3964
Yogurt	2.14 (0.27)	10	3.63 (0.56)	8	0.0163
Total daily added sugars intake^c^	53.77 (2.34) g/day		61.83 (1.37) g/day		

a *Those contributing at least 2% to total daily added sugars intake in 2011–12 (the reference year)*.

b *From linear regression analysis comparing 2017–18 to 2011–12; p ≤ 0.01 considered significant*.

c *Provided as reference to convert percentages to gram equivalents*.

Within the sweetened beverages food group, fruit drinks and soft drinks accounted for almost all of the added sugars, with fruit drinks contributing slightly more than soft drinks (Supplementary Table 1). The significantly lower contribution from fruit drinks in 2017–18 compared to 2011–12 accounted for the lower contribution from sweetened beverages over this time, while soft drinks consumption remained the same. A decline in the contribution from jams, syrups, toppings in 2017–18 compared to 2011–12 accounted for the lower contribution from sugars over this time.

### Adolescents and Teens 9–18 Years

The top two sources of added sugars intake among adolescents and teens in 2017–18 were sweetened beverages and sweet bakery products, contributing 33.5 and 14.3%, respectively, to total daily added sugars intake (Table 4). Compared to the results in younger children, the top two sources were the same; however, sweetened beverages contributed more, and sweet bakery products contributed less to added sugars intake among those 9–18 years. Candy, coffee and tea, and RTE cereals ranked number three to five, while all together nearly two-thirds (62%) of daily added sugars intake came from the top four food groups. The contribution from sweetened beverages was significantly lower in 2017–18 compared to 2011–12; however, it remained the number one source of added sugars, while RTE cereals went up in rank from number seven to number five in 2017–18.

**Table 4:** Food group sources^a^ and ranking of added sugars as a percentage of total daily added sugars intake among adolescents and teens 9–18 years, NHANES 2017–18 (*n* = 1,345) compared to NHANES 2011–12 (*n* = 1,549); values are mean (standard error) based on first day dietary recall.

**Food group**	**2017–18**		**2011–12**		***P*-value^b^**
	**% Added sugars from food group**	**Rank**	**% Added sugars from food group**	**Rank**	
Sweetened beverages	33.52 (1.27)	1	40.17 (1.60)	1	0.0011
Sweet bakery products	14.33 (1.19)	2	13.12 (0.91)	2	0.4193
Candy	7.44 (0.82)	3	5.95 (1.10)	4	0.2766
Coffee and tea	6.83 (0.86)	4	7.12 (1.20)	3	0.8466
Ready-to-eat cereals	6.79 (0.56)	5	5.17 (0.44)	7	0.0230
Other desserts	6.55 (0.89)	6	5.81 (1.08)	5	0.5976
Sugars	5.07 (0.69)	7	5.35 (1.01)	6	0.8219
Flavored milk	2.56 (0.25)	8	2.68 (0.35)	8	0.7745
Total daily added sugars intake^c^	73.13 (1.76) g/day		83.75 (2.63) g/day		

a *Those contributing at least 2% to total daily added sugars intake in 2011–12 (the reference year)*.

b *From linear regression analysis comparing 2017–18 to 2011–12; p ≤ 0.01 considered significant*.

c *Provided as reference to convert percentages to gram equivalents*.

Within the sweetened beverages food group, soft drinks and fruit drinks accounted for almost all of the added sugars, with soft drinks contributing twice as much as fruit drinks (Supplementary Table 2), in contrast to their fairly equal contributions among children. Within the coffee and tea food group, tea accounted for almost all of the added sugars. Unlike the results in children, declines in the contributions from both soft drinks and fruit drinks in 2017–18 compared to 2011–12 accounted for the lower contribution from sweetened beverages over this time. Also in 2017–18, soft drinks contributed twice as much to added sugars intake among those 9–18 years compared to those 2–8 years, and the combined contribution of soft drinks, fruit drinks, and tea was greater among those 9–18 years at 35.9% compared to 24.0% for those 2–8 years.

### Adults 19–50 Years

The top two sources of added sugars among younger adults in 2017–18 were sweetened beverages and coffee and tea, contributing 37.7 and 10.4%, respectively, to total daily added sugars intake; sweet bakery products ranked number three at 10.3%, contributing almost the same to added sugars intake as coffee and tea (Table 5). Compared to the results in adolescents and teens, sweetened beverages made a bigger contribution to added sugars intake, while sweet bakery products made a smaller contribution. Sugars and candy ranked number four and five, and 65% of added sugars intake came from the top four food groups. The only significant difference in 2017–18 compared to 2011–12 was a decline in the contribution from breads, rolls, tortillas to below 2%, but their ranking remained the same at number eight.

**Table 5:** Food group sources^a^ and ranking of added sugars as a percentage of total daily added sugars intake among adults 19–50 years, NHANES 2017–18 (*n* = 2,241) compared to NHANES 2011–12 (*n* = 2,669); values are mean (standard error) based on first day dietary recall.

**Food group**	**2017–18**		**2011–12**		***P*-value^b^**
	**% Added sugars from food group**	**Rank**	**% Added sugars from food group**	**Rank**	
Sweetened beverages	37.72 (2.15)	1	42.44 (1.47)	1	0.0702
Coffee and tea	10.36 (1.10)	2	8.89 (0.66)	3	0.2509
Sweet bakery products	10.26 (0.84)	3	12.02 (0.67)	2	0.1014
Sugars	6.68 (0.61)	4	6.70 (0.55)	4	0.9787
Candy	6.28 (0.74)	5	4.36 (0.30)	6	0.0173
Other desserts	3.55 (0.49)	6	4.46 (0.72)	5	0.2924
Ready-to-eat cereals	2.90 (0.20)	7	3.26 (0.27)	7	0.2828
Breads, rolls, tortillas	1.53 (0.13)	8	2.12 (0.16)	8	0.0042
Total daily added sugars intake^c^	72.33 (2.69) g/day		83.60 (2.29) g/day		

a *Those contributing at least 2% to total daily added sugars intake in 2011–12 (the reference year)*.

b *From linear regression analysis comparing 2017–18 to 2011–12; p ≤ 0.01 considered significant*.

c *Provided as reference to convert percentages to gram equivalents*.

Within the sweetened beverages food group, soft drinks, fruit drinks, and sport and energy drinks accounted for almost all of the added sugars, with soft drinks contributing five times as much as fruit drinks or sport and energy drinks, both of which contributed fairly equal amounts (Supplementary Table 3). The top five food categories in rank order were soft drinks, tea, sugars and honey, fruit drinks, and sport and energy drinks, and combined they accounted for almost half (46%) of added sugars intake. The contribution from soft drinks to added sugars intake was over two-fold higher than that observed in children, while similar to the results in children, and adolescents and teens, the contribution from fruit drinks declined significantly in 2017–18 compared to 2011–12.

### Adults 51–70 Years

The top two sources of added sugars among older adults in 2017–18 were sweetened beverages and sweet bakery products, contributing 28.3 and 14.6%, respectively, to total daily added sugars intake (Table 6). Sugars, coffee and tea, and candy ranked three to five, and almost two-thirds (61%) of added sugars came from the top four food groups. There was a significant decline in the contributions from RTE cereals and breads, rolls, tortillas in 2017–18 compared 2011–12, with both dropping down one rank to number eight and nine, respectively, while the contribution from breads, rolls, tortillas also fell below 2%.

**Table 6:** Food group sources^a^ and ranking of added sugars as a percentage of total daily added sugars intake among adults 51–70 years, NHANES 2017–18 (*n* = 1,776) compared to NHANES 2011–12 (*n* = 1,559); values are mean (standard error) based on first day dietary recall.

**Food group**	**2017–18**		**2011–12**		***P*-value^b^**
	**% Added sugars from food group**	**Rank**	**% Added sugars from food group**	**Rank**	
Sweetened beverages	28.29 (2.38)	1	28.76 (1.52)	1	0.8680
Sweet bakery products	14.61 (1.11)	2	14.94 (1.03)	2	0.8300
Sugars	9.18 (0.62)	3	8.72 (0.88)	3	0.6709
Coffee and tea	8.78 (1.54)	4	8.52 (1.90)	4	0.9145
Candy	6.28 (0.73)	5	7.07 (0.74)	5	0.4476
Other desserts	6.15 (0.70)	6	6.88 (0.94)	6	0.5346
Fats and oils	3.26 (0.54)	7	2.74 (0.27)	9	0.3902
Ready-to-eat cereals	2.17 (0.25)	8	3.39 (0.32)	7	0.0028
Breads, rolls, tortillas	1.91 (0.20)	9	3.16 (0.17)	8	<0.0001
Total daily added sugars intake^c^	67.85 (3.13) g/day		61.82 (2.49) g/day		

a *Those contributing at least 2% to total daily added sugars intake in 2011–12 (the reference year)*.

b *From linear regression analysis comparing 2017–18 to 2011–12; p ≤ 0.01 considered significant*.

c *Provided as reference to convert percentages to gram equivalents*.

Within the sweetened beverages food group, soft drinks accounted for approximately 75.4% of the added sugars, with the rest coming mainly from fruit drinks (Supplementary Table 4). The combined added sugars contributions from all categories of beverages, including soft drinks, fruit drinks, sport and energy drinks, and tea was 36.1%, more than twice as much as the contribution from the sweet bakery products food group.

### Adults 71+ Years

In contrast to all the other age groups, the top source of added sugars in 2017–18 among older adults 71+ years was sweet bakery products at 20.7% of total daily added sugars intake, while sweetened beverages ranked second at 17.7% (Table 7). Other desserts, sugars, and candy ranked number three to five, and almost two-thirds (63%) of added sugars intake came from the top five sources. Similar to the other adult age groups, the contribution from breads, rolls, tortillas declined significantly in 2017–18 compared to 2011–12.

**Table 7:** Food group sources^a^ and ranking of added sugars as a percentage of total daily added sugars intake among adults 71+ years, NHANES 2017–18 (*n* = 759) compared to NHANES 2011–12 (*n* = 649); values are mean (standard error) based on first day dietary recall.

**Food group**	**2017–18**		**2011–12**		***P*-value^b^**
	**% Added sugars from food group**	**Rank**	**% Added sugars from food group**	**Rank**	
Sweet bakery products	20.65 (1.35)	1	21.09 (1.62)	1	0.8348
Sweetened beverages	17.71 (1.22)	2	18.19 (1.27)	2	0.7840
Other desserts	9.46 (1.16)	3	9.54 (1.01)	4	0.9572
Sugars	7.89 (0.67)	4	11.13 (1.51)	3	0.0499
Candy	7.04 (1.63)	5	5.99 (0.79)	5	0.5624
Coffee and tea	6.15 (0.94)	6	5.82 (1.41)	6	0.8471
Ready-to-eat cereals	4.14 (0.37)	7	4.42 (0.57)	7	0.6765
Fats and oils	3.07 (0.51)	8	2.56 (0.29)	9	0.3878
Breads, rolls, tortillas	2.56 (0.23)	9	3.57 (0.22)	8	0.0013
Fruits	2.05 (0.75)	10	2.29 (0.40)	10	0.7806
Total daily added sugars intake^c^	53.81 (1.47) g/day		53.24 (2.01) g/day		

a *Those contributing at least 2% to total daily added sugars intake in 2011–12 (the reference year)*.

b *From linear regression analysis comparing 2017–18 to 2011–12; p ≤ 0.01 considered significant*.

c *Provided as reference to convert percentages to gram equivalents*.

Within the sweet bakery products food group, cakes and pies contributed the most to added sugars, followed closely by cookies and brownies, and next by a relatively small contribution from doughnuts, sweet rolls, pastries (Supplementary Table 5). Within the sweetened beverages food group, soft drinks accounted for the majority (70.1%) of added sugars; however, the added sugars contribution from soft drinks was the second lowest of all the age groups (with the lowest being among children 2–8 years). Also, fruit drinks made a very small contribution, which declined significantly in 2017–18 compared to 2011–12. A significantly lower contribution from yeast breads in 2017–18 compared to 2011–12 accounted for the lower contribution from breads, rolls, tortillas over this time.

### Gender

Using data from the combined sample (2011–18), rankings of food group sources of added sugars were similar between males and females among all ages, with only some variation in their percentage contributions. In general, males tended to have higher added sugars contributions from soft drinks compared to females, but their ranking was the same (data not shown).

### Ethnicity

Using data from the combined sample (2011–18), individuals 2–18 years had a mean intake (SE) of added sugars that ranged from a low of 48.0 g/day (1.4) among Asians to a high of 72.2 g/day (1.5) among Whites (Table 8). Among adults 19+ years, added sugars intake ranged from a low of 39.9 g/day (0.9) among Asians to a high of 78.9 g/day (1.6) among Blacks (Table 9).

**Table 8:** Sources of added sugars among children, adolescents, and teens (2–18 years) overall and from four ethnic groups, NHANES 2011–18: food categories^a^ and ranking by added sugars as a percentage of total daily added sugars intake; values based on first day dietary recall.

**Food category**	**All individuals (*****n*** **=** **10,913)^b^**		**Asian (*****n*** **=** **1,053)**		**Black (*****n*** **=** **2,736)**		**Hispanic (*****n*** **=** **3,356)**		**White (*****n*** **=** **2,998)**	
	**Mean (SE)^c^**	**Rank**	**Mean (SE)**	**Rank**	**Mean (SE)**	**Rank**	**Mean (SE)**	**Rank**	**Mean (SE)**	**Rank**
Soft drinks	18.60 (0.61)	1	10.47 (1.29)	1	15.42 (0.77)	2	20.18 (0.86)	1	19.38 (0.92)	1
Fruit drinks	11.49 (0.47)	2	7.69 (0.84)	4	20.84 (0.90)	1	12.86 (0.74)	2	8.66 (0.58)	2
Cookies and brownies	6.72 (0.24)	3	9.32 (0.78)	2	6.26 (0.38)	4	7.34 (0.45)	3	6.64 (0.39)	3
RTE^d^ cereal, higher sugar (>21.2 g/100 g)	5.54 (0.20)	4	5.40 (0.68)	6	6.07 (0.39)	5	6.39 (0.34)	4	5.00 (0.27)	7
Tea	5.18 (0.51)	5	4.24 (0.76)	9	3.60 (0.45)	10	4.41 (0.54)	7	5.93 (0.81)	4
Candy not containing chocolate	5.12 (0.25)	6	5.06 (0.53)	8	6.32 (0.41)	3	4.58 (0.33)	6	5.02 (0.38)	6
Ice cream and frozen dairy desserts	4.88 (0.25)	7	7.51 (0.96)	5	4.06 (0.28)	7	4.28 (0.33)	8	5.05 (0.39)	5
Cakes and pies	4.45 (0.32)	8	8.22 (1.42)	3	4.49 (0.64)	6	5.01 (0.66)	5	4.01 (0.49)	8
Jams, syrups, toppings	3.57 (0.20)	9	3.82 (0.60)	10	4.00 (0.39)	8	2.52 (0.26)	10	3.86 (0.34)	9
Doughnuts, sweet rolls, pastries	3.43 (0.18)	10			3.62 (0.34)	9	3.06 (0.21)	9	3.65 (0.28)	10
Candy containing chocolate	2.85 (0.24)	11	5.26 (1.05)	7					3.21 (0.38)	11
Sport and energy drinks	2.83 (0.23)	12	1.26 (0.52)	13	2.24 (0.29)	11			3.11 (0.38)	12
Total daily added sugars intake^e^ (g/day)	67.53 (0.83)		48.0 (1.4)		70.86 (1.38)		60.63 (1.29)		72.21 (1.51)	

a *Those contributing at least 2% to total daily added sugars intake among all individuals (2–18 years); empty cells represent a food category contributing <2%*.

b *Sample size for all individuals is larger than the total of the sample sizes for all ethnic groups due to missing information for some individuals (ethnicity not reported)*.

c *SE, standard error*.

d *RTE, ready-to-eat*.

e *Provided as reference to convert percentages to gram equivalents*.

**Table 9:** Sources of added sugars among adults (19+ years) overall and from four ethnic groups, NHANES 2011–18: food categories^a^ and ranking by added sugars as a percentage of total daily added sugars intake; values based on first day dietary recall.

**Food category**	**All individuals (n=19,765)^b^**		**Asian (*****n*** **=** **2,282)**		**Black (*****n*** **=** **4,528)**		**Hispanic (*****n*** **=** **4,734)**		**White (*****n*** **=** **7,490)**	
	**Mean (SE)^c^**	**Rank**	**Mean (SE)**	**Rank**	**Mean (SE)**	**Rank**	**Mean (SE)**	**Rank**	**Mean (SE)**	**Rank**
Soft drinks	25.10 (0.61)	1	16.96 (1.49)	1	25.52 (0.82)	1	31.61 (1.24)	1	23.67 (0.79)	1
Tea	8.20 (0.47)	2	6.17 (0.80)	4	8.27 (0.52)	3	5.97 (0.55)	3	8.60 (0.64)	2
Cakes and pies	5.81 (0.24)	3	5.89 (0.67)	5	6.30 (0.51)	4	5.25 (0.54)	5	5.83 (0.36)	3
Fruit drinks	5.61 (0.28)	4	4.88 (0.50)	6	12.63 (0.48)	2	7.99 (0.61)	2	3.73 (0.34)	7
Cookies and brownies	5.07 (0.16)	5	6.67 (0.53)	3	4.83 (0.24)	6	4.28 (0.25)	6	5.26 (0.24)	4
Sugars and honey	5.04 (0.16)	6	8.54 (0.53)	2	5.25 (0.29)	5	5.96 (0.30)	4	4.50 (0.22)	6
Ice cream and frozen dairy desserts	4.20 (0.19)	7	4.21 (0.36)	7	2.94 (0.20)	8	2.82 (0.24)	9	4.83 (0.26)	5
Sport and energy drinks	3.34 (0.23)	8	1.99 (0.42)	13	2.90 (0.44)	9	4.13 (0.49)	7	3.35 (0.29)	9
Candy containing chocolate	3.23 (0.17)	9	3.60 (0.40)	8	2.63 (0.21)	10			3.70 (0.25)	8
Candy not containing chocolate	2.52 (0.16)	10	2.47 (0.45)	10	3.45 (0.28)	7				
Jams, syrups, toppings	2.40 (0.14)	11	2.39 (0.38)	11	1.99 (0.15)	11	1.58 (0.17)	11	2.69 (0.20)	10
RTE^d^ cereal, higher sugar (>21.2 g/100 g)	2.33 (0.08)	12	1.92 (0.30)	15			1.92 (0.22)	10	2.44 (0.11)	11
Doughnuts, sweet rolls, pastries	2.29 (0.11)	13	2.85 (0.30)	9			3.20 (0.21)	8		
Total daily added sugars intake^e^ (g/day)	69.29 (0.84)		39.95 (0.94)		78.88 (1.59)		68.08 (1.27)		69.72 (1.16)	

a *Those contributing at least 2% to total daily added sugars intake among all individuals (19+ years); empty cells represent a food category contributing <2%*.

b *Sample size for all individuals is larger than the total of the sample sizes for all ethnic groups due to missing information for some individuals (ethnicity not reported)*.

c *SE, standard error*.

d *RTE, ready-to-eat*.

e *Provided as reference to convert percentages to gram equivalents*.

For the two overall age groups (2–18 and 19+ years), sources of added sugars were similar across ethnic groups, with sweetened beverages and sweet bakery products as the top two sources (data not shown); however, some differences across ethnic groups emerged within these food groups.

Among children, adolescents, and teens 2–18 years, soft drinks ranked number one for all ethnicities except Blacks (Table 8). Fruit drinks ranked first among Blacks, second among Hispanics and Whites, and fourth among Asians. Compared to an average of 30.1% among all ethnicities, the contribution to added sugars intake from soft drinks and fruit drinks combined was higher among Blacks (36.3%) and lower among Asians (18.2%). Within the sweet bakery products food group, cookies and brownies accounted for the majority of added sugars, while their ranking was different: second among Asians; third among Hispanics and Whites; and, fourth among Blacks.

Similar to the results in younger individuals, among adults 19+ years, soft drinks were the number one source of added sugars for all ethnicities. Compared to an average of 25.1% among all, the contribution to added sugars intake from soft drinks was higher among Hispanics (31.6%) and lower among Asians (17.0%) (Table 9). Fruit drinks varied in both ranking and added sugars contribution among ethnic groups, ranking second among Blacks and Hispanics, sixth among Asians, and seventh among Whites; and they contributed 12.6, 8.0, 4.9, and 3.7% to added sugars intake, respectively.

### Income

Using data from the combined sample (2011–2018), individuals 2–18 years had a mean intake of added sugars that was fairly consistent across PIR groups (Table 10); however, among adults 19+ years, mean intake (SE) was lowest in the high PIR group at 65.3 g/day (1.0), and highest in the low PIR group at 80.4 g/day (1.7) (Table 11).

**Table 10:** Sources of added sugars among children, adolescents, and teens (2–18 years) overall and across income strata, NHANES 2011–18: food categories^a^ and ranking by added sugars as a percentage of total daily added sugars intake; values based on first day dietary recall.

**Food category**	**All individuals (*****n*** **=** **10,913)^b^**		**Low (PIR^c^** **<** **1.35) (*****n*** **=** **4,588)**		**Medium (1.35****<****PIR****<****1.85) (*****n*** **=** **1,278)**		**High (PIR>1.85) (*****n*** **=** **4,216)**	
	**Mean (SE)^d^**	**Rank**	**Mean (SE)**	**Rank**	**Mean (SE)**	**Rank**	**Mean (SE)**	**Rank**
Soft drinks	18.60 (0.61)	1	19.68 (0.95)	1	20.43 (1.20)	1	17.38 (0.84)	1
Fruit drinks	11.49 (0.47)	2	14.01 (0.89)	2	11.58 (1.08)	2	9.56 (0.55)	2
Cookies and brownies	6.72 (0.24)	3	6.41 (0.33)	4	5.72 (0.55)	4	7.10 (0.35)	3
RTE^e^ cereal, higher sugar (>21.2 g/100 g)	5.54 (0.20)	4	6.70 (0.33)	3	6.44 (0.51)	3	4.46 (0.27)	7
Tea	5.18 (0.51)	5	5.56 (0.99)	5	5.64 (1.09)	5	4.95 (0.65)	6
Candy not containing chocolate	5.12 (0.25)	6	4.85 (0.32)	6	4.91 (0.55)	6	5.30 (0.44)	5
Ice cream and frozen dairy desserts	4.88 (0.25)	7	3.88 (0.29)	8	4.22 (0.42)	8	5.82 (0.41)	4
Cakes and pies	4.45 (0.32)	8	4.69 (0.49)	7	4.04 (0.63)	9	4.36 (0.49)	8
Jams, syrups, toppings	3.57 (0.20)	9	3.13 (0.35)	9	3.61 (0.51)	10	4.00 (0.31)	9
Doughnuts, sweet rolls, pastries	3.43 (0.18)	10	2.90 (0.25)	10	3.14 (0.51)	11	3.78 (0.29)	10
Candy containing chocolate	2.85 (0.24)	11	2.48 (0.27)	11	4.40 (1.06)	7		
Sport and energy drinks	2.83 (0.23)	12	2.40 (0.30)	12			3.24 (0.40)	11
Total daily added sugars intake^f^ (g/day)	67.53 (0.83)		66.52 (1.34)		68.02 (2.29)		68.99 (1.4)	

a *Those contributing at least 2% to total daily added sugars intake among all individuals (2–18 years); empty cells represent a food category contributing <2%*.

b *Sample size for all individuals is larger than the total of the sample sizes for all PIR groups due to missing information for some individuals (income not reported)*.

c *PIR, poverty income ratio*.

d *SE, standard error*.

e *RTE, ready-to-eat*.

f *Provided as reference to convert percentages to gram equivalents*.

**Table 11:** Sources of added sugars among adults (19+ years) overall and across income strata, NHANES 2011–18: food categories^a^ and ranking by added sugars as a percentage of total daily added sugars intake; values based on first day dietary recall.

**Food category**	**All individuals (*****n*** **=** **19,765)^b^**		**Low (PIR^c^** **<** **1.35) (*****n*** **=** **6,123)**		**Medium (1.35****<****PIR****<****.85) (*****n*** **=** **2,226)**		**High (PIR** **>** **1.85) (*****n*** **=** **9,624)**	
	**Mean (SE)^d^**	**Rank**	**Mean (SE)**	**Rank**	**Mean (SE)**	**Rank**	**Mean (SE)**	**Rank**
Soft drinks	25.10 (0.61)	1	32.07 (1.05)	1	28.21 (1.77)	1	21.36 (0.76)	1
Tea	8.20 (0.47)	2	9.56 (0.77)	2	8.27 (0.81)	2	7.76 (0.55)	2
Cakes and pies	5.81 (0.24)	3	4.77 (0.39)	5	5.28 (0.75)	4	6.46 (0.40)	3
Fruit drinks	5.61 (0.28)	4	7.06 (0.47)	3	6.46 (0.68)	3	4.73 (0.40)	6
Cookies and brownies	5.07 (0.16)	5	4.15 (0.22)	7	5.14 (0.55)	5	5.47 (0.24)	4
Sugars and honey	5.04 (0.16)	6	5.82 (0.32)	4	5.03 (0.45)	6	4.62 (0.22)	7
Ice cream and frozen dairy desserts	4.20 (0.19)	7	2.81 (0.22)	8	3.90 (0.39)	7	4.84 (0.27)	5
Sport and energy drinks	3.34 (0.23)	8	4.58 (0.54)	6	3.25 (0.54)	9	2.75 (0.23)	10
Candy containing chocolate	3.23 (0.17)	9	2.31 (0.18)	9	3.32 (0.49)	8	3.68 (0.27)	8
Candy not containing chocolate	2.52 (0.16)	10	2.24 (0.23)	10			2.74 (0.25)	11
Jams, syrups, toppings	2.40 (0.14)	11	1.74 (0.23)	13	2.00 (0.31)	11	2.83 (0.20)	9
RTE^e^ cereal, higher sugar (>21.2 g/100 g)	2.33 (0.08)	12	2.19 (0.19)	11	2.56 (0.26)	10	2.38 (0.12)	12
Doughnuts, sweet rolls, pastries	2.29 (0.11)	13	2.14 (0.18)	12			2.25 (0.12)	13
Total daily added sugars intake^f^ (g/day)	69.29 (0.84)		80.41 (1.7)		71.57 (1.82)		65.32 (0.99)	

a *Those contributing at least 2% to total daily added sugars intake among all individuals (19+ years); empty cells represent a food category contributing <2%*.

b *Sample size for all individuals is larger than the total of the sample sizes for all PIR groups due to missing information for some individuals (income not reported)*.

c *PIR, poverty income ratio*.

d *SE, standard error*.

e *RTE, ready-to-eat*.

f *Provided as reference to convert percentages to gram equivalents*.

For the two overall age groups, sources of added sugars were similar across PIR groups, with sweetened beverages and sweet bakery products as the top two sources (data not shown); however, some differences across PIR groups emerged within these and other food groups.

Among children, adolescents, and teens 2–8 years, soft drinks and fruit drinks ranked first and second among all PIR groups; however, their contributions to added sugars intake varied (Table 10). Compared to an average of 30.1% across all PIR groups, the contribution to added sugars intake from soft drinks and fruit drinks combined was higher in the low and medium PIR groups (33.7 and 32.0%, respectively) and lower in the high PIR group (26.9%). While the contribution from other sweetened beverages (tea, and sport and energy drinks combined) was highest in the low PIR group.

Compared to the results in younger individuals, among adults 19+ years, soft drinks ranked the same at number one across all three PIR groups; however, tea ranked second (Table 11). Percent contributions to added sugars intake from each beverage varied across PIR groups, with the highest contributions from soft drinks and tea in the low PIR group, and the lowest contributions from each in the high PIR group. Compared to an average of 25.1% across all PIR groups, the contribution to added sugars intake from soft drinks was higher in the low PIR group (32.1%) and lower in the high PIR group (21.4%). Fruit drinks varied in both ranking and added sugars contribution across PIR groups; they ranked third in the low and medium PIR groups and sixth in the high PIR group, and contributed 7.1 and 6.5% to added sugars intake in the low and medium PIR groups, respectively, and 4.7% in the high PIR group.

## Discussion

Using data from NHANES 2011–18, the results of this study provide a comprehensive update on the sources of added sugars in the American diet and a detailed examination of variations in added sugars sources according to selected sociodemographic factors. We estimated daily average added sugars intake at 12.7% of total calories based on NHANES 2017–18 data, which is similar to the estimate of 13% from a recent analysis using NHANES 2013–16 data (9). In looking at sources, we found the majority of added sugars in the diet came from a few sources, with sweetened beverages and sweet bakery products as the top two contributors, consistent with other studies both in the U.S. over the years 2001 to 2016 (6, 8, 9, 11, 22, 23) and in other countries (24–29). Looking further into sociodemographic factors, we also saw that the list of top added sugars sources was generally similar across age, ethnicity and PIR groups; however, there were differences in their relative contributions among these sociodemographic groups.

Our examination of added sugars sources among children, adolescents, and teens revealed distinct patterns between the younger and older age groups. Soft drinks became a bigger contributor to added sugars intake among those 9–18 years compared to younger children (2–8 years), replacing fruit drinks as the number one source and contributing twice as much to added sugars intake. The top sources of added sugars also changed with age: from two food groups, sweetened beverages (mainly fruit drinks) and sweet bakery products (cookies and brownies) among younger children, to only beverages, as sweetened beverages (mainly soft drinks and to a lesser extent, fruit drinks) and tea among adolescents and teens; and these patterns are consistent with other NHANES analyses (6, 7, 23, 30). A general shift in influence from parents and other caregivers to peers occurs in the transition from childhood to adolescence (3), and so a greater degree of parental control and family influence specifically on children's diets compared to that on the diets of adolescents and teens may explain the differences in added sugars sources that we observed in between the younger (2–8 years) and older (9–18 years) age groups. Research has shown that teens consume more sweetened beverages, and are more likely to skip breakfast and family dinners, which are behaviors that have been associated with poorer diet quality among them compared to younger children (31).

Similarly, we observed a distinct pattern in added sugars sources among adult age groups. Sweetened beverages predominated as the number one source of added sugars among adults 19–50 years and 51–70 years, mainly due to soft drinks, while sweet bakery products was the number one source among adults 71+ years. This difference is consistent with other analyses of NHANES data (23) and analyses of added sugars intakes in other countries (32, 33) and could be explained by the distinct lifestyles that characterize the working years of adulthood vs. retired older adults and the elderly. The oldest group (71+ years) represents individuals living in their retirement years, who are no longer confined by the daily routines and restrictions of working, and have more leisure time, and different social activities and contacts (34), all of which could influence dietary habits. A shift from mainly sweetened beverages to sweet bakery products as sources of added sugars may thus reflect a more leisurely routine to eating that comes with retirement, whether it is eating alone or congregating with others for socialization.

Our examination of the U.S. population stratified by ethnicity (Asians, Blacks, Hispanics, Whites) revealed that added sugars sources were similar across these groups, suggesting that ethnicity may not be an influence. Sweetened beverages and sweet bakery products were the top two sources of added sugars among all four ethnicities in both age groups, 2–18 years and 19+ years, with soft drinks being the top source; and this pattern is consistent with an analysis of earlier NHANES data (11). The one exception to soft drinks as the number one source of added sugars was observed in younger Black individuals (2–18 years), in which fruit drinks was the top source, contributing about twice as much to added sugars intake compared to the other three ethnic groups. A similar finding has been demonstrated previously using NHANES 2003–06 (11) and also more recently using NHANES 2013-16 (9), indicating a persistent pattern. Among Black adults (19+ years), fruit drinks also ranked higher (number two) and contributed more to added sugars intake compared to the other ethnic groups, suggesting fruit drinks is a common choice for this segment of the population. In contrast, added sugars intake from sweetened beverages (soft drinks and fruit drinks) was lowest among Asians of any age compared to the other three ethnic groups. This finding is supported by other research comparing added sugars sources among children (4–13 years) in the U.S., China, and Mexico, which showed Chinese children had the lowest added sugars intake overall and the lowest contributions from soft drinks and fruit drinks (35). Our findings together with those of others suggest a potential cultural basis for the observed differences in added sugars sources among Asians and the other three ethnic groups.

Similar to our findings among ethnic groups, our examination of the U.S. population stratified by income (low, medium, high PIR) revealed that added sugars sources were similar across PIR groups. Sweetened beverages and sweet bakery products were the top two sources of added sugars for all PIR groups among both younger individuals (2–18 years) and adults (19+ years). Furthermore, regardless of income, soft drinks and fruit drinks were the top contributors to added sugars intake among younger individuals, while soft drinks and tea were the top contributors among adults. However, some income differences between these two age groups emerged, with fruit drinks as the highest contributor to added sugars intake among younger individuals in the lowest PIR group, and soft drinks as the highest contributor among adults in the lowest PIR group; and this finding is consistent with a previous analysis using NHANES 2003–06, which also demonstrated a higher contribution to added sugars intake from fruit drinks among younger individuals (2–18 years) in the lowest income group (11). Research on the economics of food choices has demonstrated that added sugars are one of the lowest cost sources of dietary energy (36), and to the extent that fruit drinks and soft drinks are relatively inexpensive sources of energy, this differential may partly contribute to their greater prominence in the diets of lower income individuals. As we found fruit drinks were the top source of added sugars among Black children, income-related patterns in fruit drinks consumption may also be related to ethnicity, given Black children tend to be over-represented in the lowest income stratum (37).

Our study of added sugars sources has some strengths and limitations. One strength is our findings can be generalized to the U.S. population because our analysis is based on nationally representative data. Furthermore, NHANES data provide a rich source of information on sociodemographic variables; and by combining data from several cycles, we were able to conduct a rigorous examination of added sugars sources across segments of the population defined by ethnicity and income. We were also able to analyze added sugars sources within disaggregated age groups; for example, we separated younger individuals into two age groups, 2–8 and 9–18 years, and adults into three age groups, 19–50, 51–70, and 71+ years, allowing us to observe differences that may emerge with transitions into different life stages. Another strength of our study compared to others which required the derivation of added sugars values from algorithms (24, 25) is that our estimates of added sugars intake were based on values from the USDA FPED specific to each NHANES cycle. We also conducted and compared analyses on three different measures of added sugars sources (grams, teaspoon equivalents, and percentage of total daily added sugars intake) and results were consistent across all measures, providing confidence in the validity of our estimates.

As with any analysis of self-reported dietary intake data, our results are subject to error from recall bias and underreporting. To date, no ideal method for adjusting for underreporting effects has been identified or widely adopted (38); thus, no adjustments were applied in our analyses, consistent with the approach of other studies of added sugars sources (6, 8, 9, 11, 22–29, 39), and thereby facilitating comparisons of results across studies. Nonetheless, given that sweets and desserts are more prone to underreporting compared to other foods (40), added sugars intakes and contributions from these foods reported in our study may have been underestimated. Additionally, investigations of underreporting in NHANES samples have shown that it is more likely among older age groups (adolescents and teens vs. children, and older vs. younger adults), among Blacks compared to Whites, and among lower PIR groups (41, 42); and these differences could have contributed to the added sugars results we observed among groups stratified by these characteristics. However, with the exception of older adults, our results showed higher levels of added sugars intake among those groups in which underreporting is more likely (adolescents and teens, Blacks, and lower PIR groups), suggesting that the differences we observed were real and not simply an artifact of underreporting.

In conclusion, regardless of age, ethnicity or income, sweetened beverages and sweet bakery products were the top two sources of added sugars among the U.S. population in 2011–18. More specifically, sweetened beverages, including soft drinks and fruit drinks, as well as tea, were the largest contributors to added sugars intake. There was some variation in the types of beverages and their relative contributions across age, ethnicity, and income groups, highlighting the need to consider particular sociodemographic contexts when developing dietary guidance. For example, dietary guidance related to young children could consider their consumption of fruit drinks as a top contributor to added sugars intake, while guidance for adolescents and teens, and younger adults could consider soft drinks as the top contributor. Likewise, further examination of the factors underlying ethnic- and income-related differences in added sugars sources would contribute to a better understanding of the differences and help to target dietary guidance or other supports for healthy eating.

## Data Availability Statement

Publicly available datasets were analyzed in this study. The data used in this study are openly available in the website of the National Health and Nutrition Examination Survey: NHANES Questionnaires, Datasets, and Related Documentation; https://wwwn.cdc.gov/nchs/nhanes/Default.aspx.

## Ethics Statement

The NHANES study procedures are approved by an institutional ethics review board, and documented consent is obtained from NHANES participants.

## Author Contributions

VF, PG, MS, LR, and LD: conceptualization and writing—review and editing. VF: methodology and formal analysis. LR and LD: writing—original draft preparation. All authors have read and agreed to the published version of the manuscript.

## Conflict of Interest

LR and LD as independent consultants provide nutrition and regulatory consulting to various food manufacturers, commodity groups, and health organizations. VF as Vice President of Nutrition Impact, LLC conducts NHANES analyses for numerous members of the food, beverage, and dietary supplement industry. PG and MS are employed by The Sugar Association, Inc.
