# Supplementary material for: Sources of Added Sugars Intake Among the U.S. Population: Analysis by Selected Sociodemographic Factors Using the National Health and Nutrition Examination Survey 2011–18
Source: Front Nutr. 2021 Jun 17;8:687643. doi: 10.3389/fnut.2021.687643 (PMC8247592; doi:10.3389/fnut.2021.687643)
Supplement: Supplementary file 1 [file Table_1.DOCX]

**Table S1.** Food category sources^A^ and ranking of added sugars as a percentage of total daily added sugars intake among children 2-8 years, NHANES 2017-18 (n=914) compared to NHANES 2011-12 (n=1436); values are mean (standard error) based on first day dietary recall

| **Food Category** | **2017-18** | | **2011-12** | | **P-value**^B^ |
| --- | --- | --- | --- | --- | --- |
|  | **% Added Sugars from Food Category** | **Rank** | **% Added Sugars from Food Category** | **Rank** |  |
| Fruit drinks | 11.20 (0.98) | 1 | 16.70 (1.60) | 1 | 0.0033 |
| Cookies and brownies | 9.56 (0.88) | 2 | 7.93 (0.51) | 3 | 0.1070 |
| Soft drinks | 9.42 (1.06) | 3 | 11.59 (1.28) | 2 | 0.1917 |
| Candy not containing chocolate | 7.63 (0.99) | 4 | 5.16 (0.85) | 6 | 0.0593 |
| RTE^C^ cereal, higher sugar (>21.2g/100g) | 6.80 (0.66) | 5 | 5.91 (0.40) | 4 | 0.2516 |
| Doughnuts, sweet rolls, pastries | 5.15 (0.64) | 6 | 3.73 (0.39) | 9 | 0.0573 |
| Ice cream and frozen dairy desserts | 4.64 (0.51) | 7 | 4.71 (0.67) | 7 | 0.9292 |
| Cakes and pies | 4.47 (0.66) | 8 | 3.75 (0.65) | 8 | 0.4341 |
| Tea | 3.42 (1.08) | 9 | 2.82 (1.02) | 12 | 0.6885 |
| Jams, syrups, toppings | 2.95 (0.31) | 10 | 5.24 (0.94) | 5 | 0.0201 |
| Gelatins, ices, sorbets | 2.03 (0.51) | 11 | 2.14 (0.52) | 13 | 0.8791 |
| Flavored milk, reduced fat | 1.81 (0.30) | 12 | 2.84 (0.48) | 11 | 0.0666 |
| Yogurt, regular | 1.76 (0.25) | 13 | 3.63 (0.56) | 10 | 0.0024 |
| Total Daily Added Sugars Intake^D^ | 53.77 (2.34) g/day | | 61.83 (1.37) g/day | |  |

^A^ Those contributing at least 2% to total daily added sugars intake in 2011-12 (the reference year)

^B^ From linear regression analysis comparing 2017-18 to 2011-12; p<0.01 considered significant

^C^ RTE, ready-to-eat

^D^ Provided as reference to convert percentages to gram equivalents

**Table S2.** Food category sources^A^ and ranking of added sugars as a percentage of total daily added sugars intake among adolescents and teens 9-18 years, NHANES 2017-18 (n=1345) compared to NHANES 2011-12 (n=1549); values are mean (standard error) based on first day dietary recall

| **Food Category** | **2017-18** | | **2011-12** | | **P-value**^B^ |
| --- | --- | --- | --- | --- | --- |
|  | **% Added Sugars from Food Category** | **Rank** | **% Added Sugars from Food Category** | **Rank** |  |
| Soft drinks | 20.61 (1.43) | 1 | 23.64 (1.66) | 1 | 0.1674 |
| Fruit drinks | 9.10 (0.97) | 2 | 12.84 (1.53) | 2 | 0.0389 |
| Cookies and brownies | 6.59 (0.60) | 3 | 5.10 (0.54) | 4 | 0.0639 |
| Tea | 6.19 (0.85) | 4 | 6.63 (1.24) | 3 | 0.7697 |
| RTE^C^ cereal, higher sugar (>21.2g/100g) | 6.13 (0.66) | 5 | 4.52 (0.45) | 6 | 0.0454 |
| Ice cream and frozen dairy desserts | 5.74 (0.97) | 6 | 4.11 (0.53) | 7 | 0.1412 |
| Cakes and pies | 4.92 (1.18) | 7 | 4.97 (0.69) | 5 | 0.9701 |
| Candy not containing chocolate | 4.68 (0.62) | 8 | 2.99 (0.56) | 11 | 0.0441 |
| Jams, syrups, toppings | 3.44 (0.51) | 9 | 3.52 (0.78) | 9 | 0.9326 |
| Sport and energy drinks | 3.26 (0.45) | 10 | 3.64 (0.61) | 8 | 0.6196 |
| Doughnuts, sweet rolls, pastries | 2.81 (0.33) | 11 | 3.04 (0.32) | 10 | 0.6208 |
| Candy containing chocolate | 2.77 (0.56) | 12 | 2.96 (0.65) | 12 | 0.8175 |
| Total Daily Added Sugars Intake^D^ | 73.13 (1.76) g/day | | 83.75 (2.63) g/day | |  |

^A^ Those contributing at least 2% to total daily added sugars intake in 2011-12 (the reference year)

^B^ From linear regression analysis comparing 2017-18 to 2011-12; p<0.01 considered significant

^C^ RTE, ready-to-eat

^D^ Provided as reference to convert percentages to gram equivalents

**Table S3.** Food category sources^A^ and ranking of added sugars as a percentage of total daily added sugars intake among adults 19-50 years, NHANES 2017-18 (n=2241) compared to NHANES 2011-12 (n=2669); values are mean (standard error) based on first day dietary recall

| **Food Category** | **2017-18** | | **2011-12** | | **P-value**^B^ |
| --- | --- | --- | --- | --- | --- |
|  | **% Added Sugars from Food Category** | **Rank** | **% Added Sugars from Food Category** | **Rank** |  |
| Soft drinks | 27.80 (2.14) | 1 | 28.78 (1.50) | 1 | 0.7068 |
| Tea | 8.41 (0.96) | 2 | 7.79 (0.69) | 3 | 0.5973 |
| Sugars and honey | 4.97 (0.50) | 3 | 4.27 (0.31) | 7 | 0.2347 |
| Fruit drinks | 4.68 (0.40) | 4 | 8.98 (1.23) | 2 | 0.0009 |
| Sport and energy drinks | 4.60 (0.48) | 5 | 4.52 (0.88) | 5 | 0.9362 |
| Cookies and brownies | 4.21 (0.44) | 6 | 4.40 (0.34) | 6 | 0.7322 |
| Cakes and pies | 4.17 (0.57) | 7 | 5.62 (0.38) | 4 | 0.0335 |
| Candy containing chocolate | 3.18 (0.48) | 8 | 2.44 (0.23) | 10 | 0.1605 |
| Ice cream and frozen dairy desserts | 3.05 (0.43) | 9 | 3.33 (0.54) | 8 | 0.6829 |
| RTE^C^ cereal, higher sugar (>21.2g/100g) | 2.42 (0.21) | 10 | 2.65 (0.24) | 9 | 0.4729 |
| Jams, syrups, toppings | 1.71 (0.20) | 11 | 2.43 (0.38) | 11 | 0.0948 |
| Total Daily Added Sugars Intake^D^ | 72.33 (2.69) g/day | | 83.60 (2.29) g/day | |  |

^A^ Those contributing at least 2% to total daily added sugars intake in 2011-12 (the reference year)

^B^ From linear regression analysis comparing 2017-18 to 2011-12; p<0.01 considered significant

^C^ RTE, ready-to-eat

^D^ Provided as reference to convert percentages to gram equivalents

**Table S4.**  Food category sources^A^ and ranking of added sugars as a percentage of total daily added sugars intake among adults 51-70 years, NHANES 2017-18 (n=1776) compared to NHANES 2011-12 (n=1559); values are mean (standard error) based on first day dietary recall

| **Food Category** | **2017-18** | | **2011-12** | | **P-value**^B^ |
| --- | --- | --- | --- | --- | --- |
|  | **% Added Sugars from Food Category** | **Rank** | **% Added Sugars from Food Category** | **Rank** |  |
| Soft drinks | 21.33 (2.26) | 1 | 19.31 (1.29) | 1 | 0.4356 |
| Tea | 8.16 (1.55) | 2 | 7.90 (1.92) | 2 | 0.9139 |
| Sugars and honey | 6.38 (0.45) | 3 | 5.30 (0.48) | 7 | 0.1021 |
| Cakes and pies | 6.00 (0.94) | 4 | 7.39 (1.30) | 3 | 0.3861 |
| Cookies and brownies | 5.69 (0.70) | 5 | 5.47 (0.50) | 5 | 0.7972 |
| Ice cream and frozen dairy desserts | 5.19 (0.63) | 6 | 5.40 (0.78) | 6 | 0.8363 |
| Fruit drinks | 4.83 (0.73) | 7 | 6.99 (0.91) | 4 | 0.0628 |
| Candy containing chocolate | 3.45 (0.65) | 8 | 4.42 (0.84) | 8 | 0.3586 |
| Doughnuts, sweet rolls, pastries | 2.93 (0.55) | 9 | 2.08 (0.45) | 13 | 0.2349 |
| Candy not containing chocolate | 2.83 (0.44) | 10 | 2.64 (0.45) | 10 | 0.7612 |
| Jams, syrups, toppings | 2.80 (0.44) | 11 | 3.42 (0.68) | 9 | 0.4403 |
| Sport and energy drinks | 1.77 (0.49) | 12 | 2.21 (0.74) | 12 | 0.6235 |
| RTE^C^ cereal, higher sugar (>21.2g/100g) | 1.71 (0.20) | 13 | 2.43 (0.28) | 11 | 0.0355 |
| Total Daily Added Sugars Intake^D^ | 67.85 (3.13) g/day | | 61.82 (2.49) g/day | |  |

^A^ Those contributing at least 2% to total daily added sugars intake in 2011-12 (the reference year)

^B^ From linear regression analysis comparing 2017-18 to 2011-12; p<0.01 considered significant

^C^ RTE, ready-to-eat

^D^ Provided as reference to convert percentages to gram equivalents

**Table S5.** Food category sources^A^ and ranking of added sugars as a percentage of total daily added sugars intake among adults 71+ years, NHANES 2017-18 (n=759) compared to NHANES 2011-12 (n=649); values are mean (standard error) based on first day dietary recall

| **Food Category** | **2017-18** | | **2011-12** | | **P-value**^B^ |
| --- | --- | --- | --- | --- | --- |
|  | **% Added Sugars from Food Category** | **Rank** | **% Added Sugars from Food Category** | **Rank** |  |
| Soft drinks | 12.50 (1.39) | 1 | 9.44 (1.12) | 2 | 0.0872 |
| Cakes and pies | 9.37 (1.26) | 2 | 8.83 (1.20) | 3 | 0.7568 |
| Cookies and brownies | 8.55 (0.90) | 3 | 9.47 (0.91) | 1 | 0.4707 |
| Ice cream and frozen dairy desserts | 8.00 (0.98) | 4 | 7.21 (0.92) | 4 | 0.5540 |
| Tea | 5.59 (1.00) | 5 | 5.53 (1.41) | 7 | 0.9709 |
| Sugars and honey | 4.84 (0.56) | 6 | 5.88 (1.07) | 6 | 0.3910 |
| Candy containing chocolate | 4.08 (1.34) | 7 | 4.16 (0.64) | 9 | 0.9572 |
| RTE^C^ cereal, higher sugar (>21.2g/100g) | 3.11 (0.30) | 8 | 3.09 (0.43) | 10 | 0.9634 |
| Fruit drinks | 3.06 (0.45) | 9 | 6.26 (1.06) | 5 | 0.0054 |
| Jams, syrups, toppings | 3.04 (0.67) | 10 | 5.25 (0.82) | 8 | 0.0370 |
| Doughnuts, sweet rolls, pastries | 2.73 (0.63) | 11 | 2.79 (0.46) | 11 | 0.9409 |
| Yeast breads | 1.75 (0.17) | 12 | 2.61 (0.22) | 12 | 0.0022 |
| Total Daily Added Sugars Intake^D^ | 53.81 (1.47) g/day | | 53.24 (2.01) g/day | |  |

^A^ Those contributing at least 2% to total daily added sugars intake in 2011-12 (the reference year)

^B^ From linear regression analysis comparing 2017-18 to 2011-12; p<0.01 considered significant

^C^ RTE, ready-to-eat

^D^ Provided as reference to convert percentages to gram equivalents
